# Development and Validation of an HPLC-MS/MS Method for the Early Diagnosis of Aspergillosis

**DOI:** 10.1371/journal.pone.0092851

**Published:** 2014-04-01

**Authors:** Letícia B. Cerqueira, Thais M. G. de Francisco, João C. Gasparetto, Francinete R. Campos, Roberto Pontarolo

**Affiliations:** Department of Pharmacy, Federal University of Paraná, Curitiba, Paraná, Brazil; Albert Einstein College of Medicine, United States of America

## Abstract

Invasive aspergillosis is an opportunistic infection that is mainly caused by *Aspergillus fumigatus*, which is known to produce several secondary metabolites, including gliotoxin, the most abundant metabolite produced during hyphal growth. The diagnosis of invasive aspergillosis is often made late in the infection because of the lack of reliable and feasible diagnostic techniques; therefore, early detection is critical to begin treatment and avoid more serious complications. The present work reports the development and validation of an HPLC-MS/MS method for the detection of gliotoxin in the serum of patients with suspected aspergillosis. Chromatographic separation was achieved using an XBridge C18 column (150×2.1 mm id; 5 mm particle size) maintained at 25°C with the corresponding guard column (XBridge C18, 10×2.1 mm id, 5 mm particle size). The mobile phase was composed of a gradient of water and acetonitrile/water (95∶5 *v/v*), both containing 1 mM ammonium formate with a flow rate of 0.45 mL min^−1^. Data from the validation studies demonstrate that this new method is highly sensitive, selective, linear, precise, accurate and free from matrix interference. The developed method was successfully applied to samples from patients suspected of having aspergillosis. Therefore, the developed method has considerable potential as a diagnostic technique for aspergillosis.

## Introduction

Opportunistic invasive fungal infections are a major cause of morbidity and mortality following bone marrow transplantation. After surgery, patients begin treatment with immunosuppressive drugs to prevent organ rejection, which in turn decreases the immune response of these patients, making them more susceptible to infection by opportunistic pathogens commonly found in the environment [1]. *Aspergillus fumigatus* accounts for approximately 90% of invasive aspergilloses diagnoses [2].

The diagnosis of aspergillosis is complex, particularly in immunocompromised patients. Signs and symptoms are non-specific, colonization is difficult to distinguish from invasive disease, blood cultures are commonly negative and patients are often unable to undergo invasive diagnostic procedures. This situation has led to the strategy of initiating empirical therapy in high-risk patients [3]. Typically, the diagnosis is based on histopathology or culture [4]. However, because the symptoms are similar to those of other diseases, the start of the correct treatment is delayed, reducing its effectiveness and, in some cases, leading to death [5]. Many studies have been published reporting conventional techniques of diagnosing *A. fumigatus*, including microbiological, histological and radiological analyses. However, these techniques present disadvantages, such as low sensitivity and specificity, high rates of false-positive results and a long time required to confirm the diagnosis [6]. Biopsies are also commonly used to diagnose *A. fumigatus*; however, invasive procedures are not encouraged because of the weakened state of the patient [7]–[9]. The diagnosis of *A. fumigatus* has been accomplished using enzyme immunoassay tests against the galactomannan antigen [4]. Although this immunoassay looks promising, studies have demonstrated that the quantification of galactomannans by this technique can promote false-positive results because of the administration of antibiotics and can result from infections by fungi other than *Aspergillus*. Other tests used to diagnose invasive aspergillosis include (1→3)-β-D-glucan and polymerase chain reaction (PCR); however, these methods have not yet been evaluated for their validity in diagnosing this condition [4].

Several reports suggest that the suppression of the immune function of the host by mycotoxins (secondary metabolites released by fungi) is one of the possible mechanisms underlying why the fungus is not counteracted by the immune system [2], [11]. Gliotoxin is one of the most toxic metabolites produced during the growth of several species of fungi, including *Aspergillus fumigatus*, *Eurotium chevalieri*, *Gliocladium fimbriatum* as well as *Penicillium* and *Trichoderma* spp. This toxicity is the result of the intramolecular disulfide bridge, which is the subject of many investigations of its structure and activity [12]. Sutton and coworkers (1996) demonstrated that the immune suppression produced by gliotoxin may promote the establishment of invasive aspergillosis [13]. Studies conducted in the lungs of rats with induced invasive aspergillosis showed that the presence of gliotoxin diminished the recognition of the fungus by the cells of the immune system [14]. Gliotoxin was also found in mice and humans with aspergillosis [14]. Based on these findings, it is evident that detection of gliotoxin by a sensitive, precise and accurate method may be an option for diagnosing invasive aspergillosis.

Among the existing methods for the detection and quantification of gliotoxin, a semi-quantitative bioassay based on an automated microplate-reader operated at 630 nm was developed by Grovel and coworkers in 2006. This bioassay allowed for the rapid screening of samples for gliotoxin concentration in extracts with limits of detection of approximately 18 to 20 ng [15]. Gliotoxin was also measured by LC-MS-MS in the sera of mice (36.5±30.28 ng mL^−1^) with experimentally induced invasive aspergillosis (IA) and in the sera of cancer patients with documented (proven or probable) IA (65–785 ng mL^−1^) [14]. None of the described methods have precise materials and methods, making them difficult to reproduce. Additionally, none have been fully validated according to the modern worldwide regulations; thus, the reliability of their results cannot be ensured.

The aim of this work is the development and validation of a new method based on high-performance liquid chromatography tandem mass spectrometry (HPLC-MS/MS) to detect gliotoxin in human serum for the early diagnosis of invasive aspergillosis.

## Materials and Methods

### 1. Samples, Chemicals and Reagents

**Figure 1:**
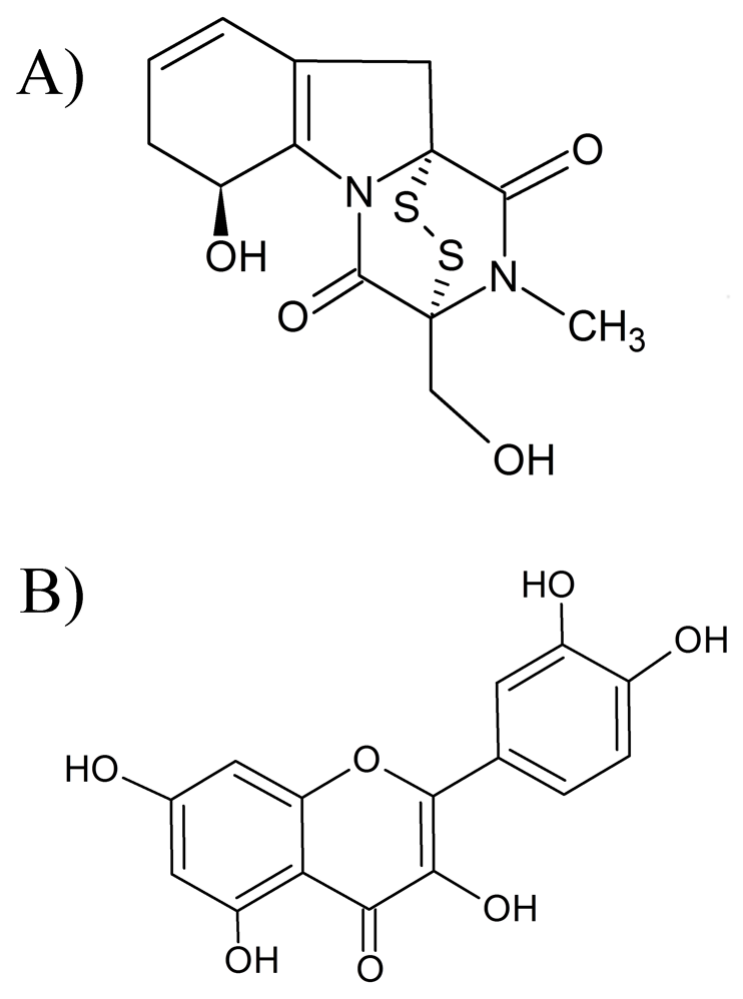
Chemical structures of (A) gliotoxin, (B) quercetin (IS). The structures were draw in the ChemSketch 11.0 software [16].

A pool of blank, hemolyzed and lipemic serum was obtained from healthy volunteers and were used in validation experiments (Section 6). Serum samples from patients hospitalized after bone marrow transplantation and patients undergoing chemotherapy were provided by the Hospital de Clinicas – UFPR and used for method application (Section 8). Methanol and acetonitrile (HPLC grade) were purchased from Tedia (Fairfield, Ohio, USA), and formic acid (88%) was obtained from J. T. Baker Chemicals B.V. (Deventer, The Netherlands). Ammonium formate (97%) was purchased from Acros Organics (New Jersey, USA). Ultrapure water was obtained using a Milli-Q™ purification system from the Millipore Corporation (Bedford, USA). Ether (99%), ethyl acetate (99.8%), a gliotoxin standard (99.0%) and the internal standard (IS) quercetin (99.0%) were purchased from Sigma Aldrich (St. Louis, USA). The structure of each metabolite is shown in Figure 1.

### 2. Standard solutions

Stock solutions of gliotoxin and IS were prepared separately in methanol at concentrations of 1 mg mL^−1^. All stock solutions were stored at 4°C. Working standard solutions were freshly prepared from these stock solutions as needed for each experiment via appropriate dilution with acetonitrile/water (98∶2 *v/v*).

### 3. HPLC-MS/MS instrumentation and conditions

HPLC-MS/MS analyses were performed using an Agilent 1200 HPLC System (Wilmington, USA) consisting of a G1312B binary pump, a G1379B degasser and a G1316B column oven. The HPLC was connected to a CTC Sample Manager (Model 2777, Waters Corporation, Milford, USA). The HPLC system was coupled to an Applied Biosystems MDS Sciex API 3200 Triple Quadrupole Mass Spectrometer (Toronto, Canada) equipped with a Harvard 22 Dual Model syringe pump (Harvard Apparatus, South Natick, USA) and an electrospray ionization (ESI) source. The mobile phase consisted of a gradient of water (A) and acetonitrile/water 95∶5 *v/v* (B), both containing 1 mM ammonium formate, eluted using the following gradient program: t_0 to 0.10 min_: A = 65%; t_0.11 to 0.70 min_: A = 0%; t_0.71 to 4.0 min_: A = 65%. The flow rate was 0.45 mL min^−1^. The analytical separations were achieved on an XBridge Shield C18 150×2.1 mm (5 µm particle size) column coupled with an XBridge C18 10×2.1 mm (5 µm particle size) guard column (Waters Corporation, Milford, USA). The injection volume was 20 µL, and the column temperature was maintained at 45°C. Data acquisition was performed with the MS Workstation using Analyst 1.4 software (ABI/Sciex). The ESI source was operated in the negative ion mode for monitoring gliotoxin and IS. Quantification was performed in the Multiple Reaction Monitoring (MRM) mode while maintaining the dwell time at 450 ms. The ion source parameters were as follows: curtain gas (CUR), 10 psi; collision gas (CAD), 6 psi; ion spray voltage (ISV), −4500 V; nebulizer gas (GS1), 45 psi; turbo gas (GS2), 45 psi and temperature, 450°C. The ion transitions and individual compound parameters, including the declustering potential (DP), entrance potential (EP), collision cell entrance potential (CEP), collision energy (CE) and cell exit potential (CXP), are shown in Table 1. The high-purity nitrogen and zero-grade air used as the CUR, GS1, GS2 and CAD gases were produced using a high-purity nitrogen generator from PEAK Scientific Instruments (Chicago, USA).

**Table 1:** Compound-dependent parameters and ion transitions of gliotoxin and internal standard (IS) used for the quantification.

Compound	Molecular ion (*m/z*)	Ion transition (*m/z*)	CE^a^(eV)	CEP^b^(V)	CXP^c^(V)	DP^d^(V)	EP^e^(V)
Gliotoxin	324.9	324.9→261.2	−12	−20	−6	−20	−6
		324.9→243.1	−20	−20	−4	−20	−6
		300.8→151.0	−30	−16	−2	−35	−6
		300.8→179.0	−26	−16	−4	−35	−4

a CE, collision energy;

b CEP, collision cell entrance potential;

c CXP, cell exit potential;

d DP, declustering potential;

e EP, entrance potential.

### 4. Evaluation of different sample extraction procedures

To determine the best extraction procedures for gliotoxin and IS in human serum, several methods were tested, including protein precipitation, liquid-liquid extraction and solid phase extraction. The analyses were performed using six replicates. For each compound and each extraction procedure, the sensitivity and accuracy of the method were evaluated using the peak area, and the reproducibility was determined by the relative standard deviation (RSD%) of the peak area.

#### 4.1. Serum fortification

A 200 µL aliquot of blank serum (serum free of analytes and IS) was transferred into a 2 mL plastic centrifuge tube. The serum samples were each spiked with 50 µL of the working standard solution and 50 µL of the working IS solution to obtain final concentrations of 250 ng mL^−1^ of gliotoxin and 5.0 ng mL^−1^ of IS. The samples were vortexed for 3 min and then subjected to different extraction procedures.

#### 4.2. Extraction procedures

*4.2.1 Protein precipitation:* A 1.2 mL aliquot of acetonitrile or methanol was added to the centrifuge tubes containing the homogeneous spiked serum (Section 4.1). The samples were vortexed for 3 min and then centrifuged at 14000 rpm for 10 min at 25°C (Eppendorf 5810R, Hamburg, Germany). The total volume of the supernatant was poured into a new plastic centrifuge tube and evaporated until dry in a sample concentrator (25°C) (CentriVap Labconco, Kansas City, USA). The samples were resuspended in 200 µL of acetonitrile/water (98∶2 *v/v*) containing 0.1% formic acid and vortexed for 3 min.

*4.2.2 Solid phase extraction:* The total volume of the homogeneous spiked serum (Section 4.1) was transferred to an Oasis HLB Sample Extraction Cartridge™ (1cc, 30 mg) previously conditioned with methanol and ultrapure water. The extraction cartridge was flushed twice with water (1 mL each), and the retained analytes were recovered by washing the cartridges with 1 mL of pure acetonitrile. The eluate was evaporated until dry in a sample concentrator (25°C) and redissolved in 200 µL of acetonitrile/water (98∶2 *v/v*) containing 0.1% formic acid by vortexing for 3 min.

**Figure 2:**
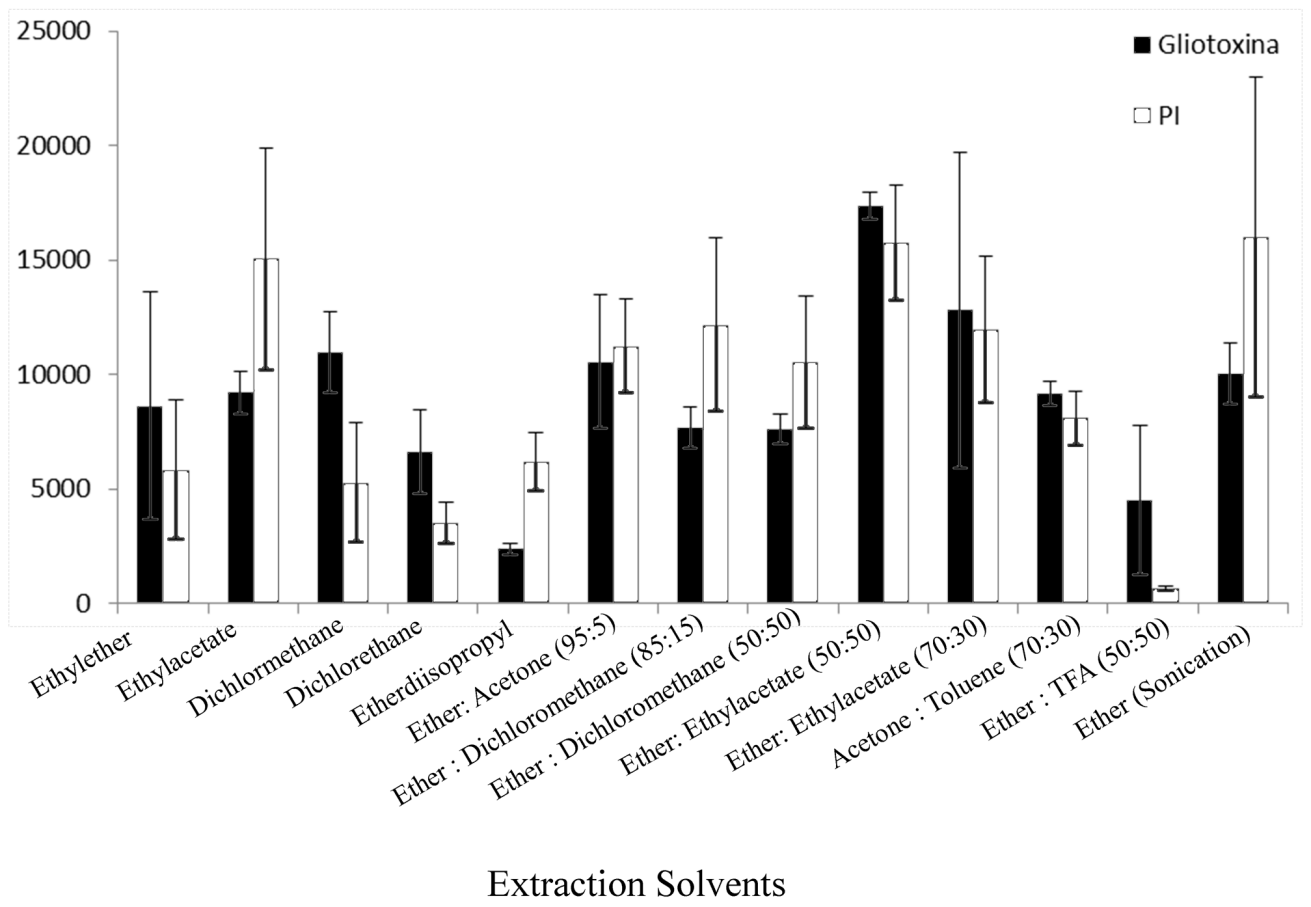
Recoveries of gliotoxin and quercetin (IS) using liquid-liquid extraction with different solvents. Data: peak area obtained with blank serum spiked with standards of gliotoxin (250.0 ng mL^−1^) and the IS quercetin (5.0 ng mL^−1^); TFA: trifluoroacetic acid; error bars: expressed as the standard deviation of six replicate injections.

*4.2.3 Liquid-liquid extraction:* A 1.2 mL aliquot of one of the various extraction solvents (toluene, diethyl ether, ethyl acetate, dichloromethane, dichloroethane, diisopropyl ether, acetone and mixtures of these solvents in the proportions given in Figure 2) was added to each individual centrifuge tube, each containing the homogeneous spiked serum (Section 4.1). The samples were vortexed for 3 min and then centrifuged at 14000 rpm for 10 min at 25°C. A 1.2 mL aliquot of each sample supernatant was transferred to a new plastic centrifuge tube and evaporated until dry in a sample concentrator (25°C). The samples were resuspended in 200 µL of acetonitrile/water (98∶2 *v/v*) containing 0.1% formic acid and vortexed for 3 min.

### 5. Sample preparation

All frozen (−40°C) human serum samples from volunteers were thawed at room temperature, and a 200 µL aliquot was transferred into each 2 mL plastic centrifuge tube. The samples were spiked with a 50 µL aliquot of acetonitrile/water (98∶2 *v/v*) and 50 µL of the working IS solution (5.0 ng mL^−1^). The spiked samples were vortexed for 3 min, and an aliquot of 1.2 mL of diethyl ether-ethyl acetate (50∶50 *v/v*) was added to each plastic tube. The samples were vortexed for 3 min and then centrifuged for 10 min (14000 rpm at room temperature). A 1.0 mL aliquot of each sample supernatant was transferred to a new plastic centrifuge tube and evaporated in a sample concentrator (25°C). The samples were resuspended in 200 µL of acetonitrile/water (98∶2 *v/v*) containing 0.1% formic acid by vortexing for 3 min.

### 6. Validation of the analytical method

The proposed method was validated by determining the selectivity, limits of detection (LOD) and quantification (LOQ), linearity, precision (intra- and inter-day) and accuracy, as described in the Food and Drug Administration Bioanalytical Method Validation guideline [17].

#### 6.1. Limit of detection (LOD) and lower limit of quantification (LLOQ)

The LOD and LLOQ were estimated from the signal-to-noise ratio. To conduct this study, blank serum was spiked in triplicate with the working standard solution to a final concentration of 100 ng mL^−1^ of gliotoxin. The spiked samples were diluted in series with blank serum and injected until the smallest detectable peak was obtained. The LOD was estimated at a signal-to-noise ratio of 3∶1, and the LLOQ was estimated at a signal-to-noise ratio of at least 10∶1, until the desired precision was obtained (relative standard deviation <20%).

#### 6.2. Selectivity

To evaluate the selectivity, hemolyzed serum, lipemic serum and four lots of blank serum were spiked with 100 µL of acetonitrile/water (98∶2 v/v). The samples were vortexed for 3 min and then subjected to a liquid-liquid extraction procedure, as described in Section 4.2.3, using diethyl ether-ethyl acetate (50∶50 v/v). The samples were injected into the HPLC-MS/MS, and the chromatograms obtained were compared with the chromatograms from serum spiked at the LLOQ concentrations of gliotoxin (10 ng mL^−1^) and IS (5 ng mL^−1^). No significant interfering peaks should be observed at the retention times of the analyte and internal standard.

#### 6.3. Quality control samples, calibration standards and linearity

*6.3.1. Quality control samples:* Quality control (QC) samples were prepared by spiking 200 µL of blank serum with 50 µL of the analyte solution and 50 µL of the IS solution to obtain final concentrations of 10, 30, 70 and 110 ng mL^−1^of gliotoxin. These values correspond to the lower limit of quantification (LLOQ), low quality control (LQC), medium quality control (MQC) and high quality control (HQC), respectively, with each level containing 5 ng mL^−1^ of IS. Spiked samples were subjected to an extraction procedure as described in Section 5.

*6.3.2. Calibration standards and linearity:* The linearity of the assay was investigated using the internal standardization method at seven concentration levels for each compound. Calibration curves were prepared in triplicate on three different days by spiking 200 µL of blank serum with 50 µL of IS solution and 50 µL of analyte solution to obtain the following concentrations: 10, 20, 40, 60, 80, 100 and 120 ng mL^−1^ of gliotoxin, with each level containing 5 ng mL^−1^ of IS. After spiking, the samples were subjected to the extraction procedure, as described in Section 4.2.3, using diethyl ether-ethyl acetate (50∶50 *v/v*) as the extraction solvent. A calibration curve was generated to confirm the linear relationship between the analyte peak area/IS peak area versus the analyte concentration/IS concentration. The slope, intercept and regression coefficient (r) were calculated by a weighted (1/x) linear regression. Variation of less than 15% in accuracy and precision at each level should be expected, except at the LLOQ, where no variations exceeding 20% should be observed. The linear correlation coefficient (r) must also be equal to or greater than 0.98 [17], [18].

#### 6.4. Matrix effect

To evaluate the matrix effect, four aliquots of 200 µL of blank serum, two aliquots of 200 µL of lipemic serum and two aliquots of 200 µL of hemolyzed serum from different lots were initially spiked with 100 µL of acetonitrile/water (98∶2 v/v). The spiked samples were vortexed for 3 min, and 1.2 mL aliquot of a mixture of diethyl ether-ethyl acetate (50∶50 v/v) was added to each tube. The samples were vortexed for 3 min and then centrifuged for 10 min (14000 rpm at 25°C). A 1.0 mL aliquot of each sample supernatant was transferred to a new plastic centrifuge tube and evaporated (25°C) in a sample concentrator. The samples were resuspended in 100 µL of acetonitrile/water (98∶2 v/v), 50 µL of the working standard solution and 50 µL of the working IS solution to obtain the same concentration levels as the LQC and HQC samples (Section 6.3.1). For each concentration level, the normalized matrix effect (NME) of the responses of the analytes in serum and in solution was calculated (analyte response in matrix/IS response in matrix versus analyte response in solution/IS response in solution). Variations of less than 15% relative to the NME calculated for all concentration levels of each analyte indicate that the matrix effect is not significant.

#### 6.5. Carryover test

To perform a carryover test, samples of 120 ng mL^−1^ of gliotoxin containing IS at 5 ng mL^−1^ were prepared and injected alternately with blank serum into the HPLC-MS/MS system. No significant interfering peaks were observed in the blank serum chromatogram at the retention times of the analytes or the internal standard.

#### 6.6. Accuracy, precision and sample dilution

Intra- and inter-day accuracy and precision were demonstrated by preparing QC samples (Section 6.3.1) in five replicates on three consecutive days at the following concentrations: 10, 30, 70, 110 and 70 ng mL^−1^ of gliotoxin, with each level containing 5 ng mL^−1^ of IS. Intra- and inter-day precision values were estimated using the RSD% at each concentration level, while the accuracy values were estimated by calculating the difference between the calculated and theoretical concentrations. For both accuracy and precision, variation must be within 15%, except at the LLOQ, where the variation should not exceed 20%.

The dilution test was performed to demonstrate that samples subjected to dilution have the same precision and accuracy as undiluted samples. The test was performed in five replicates by spiking blank serum with standard solutions to obtain a final concentration of 700 ng mL^−1^ of gliotoxin. The samples were diluted tenfold with blank serum, processed and analyzed. The diluted concentrations were compared with the original concentrations using the relative error (RE%) and the precision (RSD%) was also evaluated.

#### 6.7. Recovery

To examine the recovery, a batch of five replicate QC samples spiked with standards before extraction (set 1) were prepared as described in Section 6.3.1 at concentrations of 70.0 ng mL^−1^gliotoxin and 5.0 ng mL^−1^IS. Another batch spiked with standards after extraction (set 2) was prepared by spiking 200 µL of blank serum with 100 µL of acetonitrile/water (98∶2 v/v) and extracting the samples with diethyl ether-ethyl acetate (50∶50 v/v) (Section 4.2.3). The samples were resuspended in 100 µL of acetonitrile/water (98∶2 v/v), 50 µL of IS and 50 µL of analyte standard solution, to generate solutions with the same concentrations as the QC samples. The recovery was determined by comparing the peak areas obtained from set 1 with the peak areas obtained from set 2.

#### 6.8. Stability study

The stability of gliotoxin and IS was tested using six replicates under a variety of storage and handling conditions. Short-term stability was assayed by spiking 200 µL of blank serum with 50 µL of analyte solution and 50 µL of IS solution to obtain final concentrations of 30 and 110 ng mL^−1^ for gliotoxin and 5 ng mL^−1^ for IS (each level). The samples were extracted with diethyl ether-ethyl acetate (50∶50 v/v) (Section 4.2.3) and then kept at room temperature for 6 h. The short-term stability was evaluated by comparing the mean recoveries of the analyte and IS obtained from stored samples to the mean recoveries obtained from freshly prepared samples.

Freeze-thawed, post-preparative and long-term stability assay samples were prepared and evaluated similarly to the short-term stability samples. However, the freeze thawing assay was performed after subjecting the samples to three freeze-thaw cycles (one freeze-thaw cycle per day, three consecutive days), and the post-preparative assay samples were stored for 24 hours in the Sample Manager (4°C, transparent vial). The long-term stability of human serum stored at −40°C was assessed at the end of the study (30 days).

The stability of stock solutions (1 mg mL^−1^ of gliotoxin and IS) was evaluated after 24 and 48 hours of storage in a refrigerator (4°C, amber bottle) and after 30 days of storage in a freezer (−40°C). The stored solutions were diluted with acetonitrile/water (98∶2 *v/v*) to obtain final concentrations of 30.0 and 110.0 ng mL^−1^ of gliotoxin and 5.0 ng mL^−1^ of IS. The mean recoveries of the analyte and IS obtained with the stored solutions were compared with the mean recoveries obtained with freshly prepared solutions at the same concentration levels. The stability of the working standard solutions was assessed following a procedure similar to the one previously described for stock solution stability, except that the samples were stored for 6 hours at room temperature.

### 7. Ethics statement regarding human samples

All studies involving human serum samples were approved by the Ethics Committee of Federal University of Paraná under number 10857012.0.0000.0102 of the Certificate of Presentation for Ethics Appreciation (CAAE). The volunteers (patients suspected of having opportunistic infections after bone marrow transplantation or who were undergoing chemotherapy) were informed that the blood samples collected for routine analysis would be used for new research. All volunteers gave written informed consent to participate in the study.

### 8. Application of the developed method

The developed method was applied to serum samples provided by the Hospital de Clínicas-UFPR. The provided samples were stored in a freezer at −40°C until analysis. All samples were identified by the hospital as negative or positive according to the results of the ELISA for galactomannans (Platelia™ *Aspergillus*, BioRad). The developed HPLC-MS/MS method was applied to 30 samples, including 10 that were positive and 20 that were negative for the presence of galactomannans. The results of the ELISA provided by the hospital were used as a reference to compare the results obtained by the developed HPLC-MS/MS method.

## Results and Discussion

### 1. Method development

All of the MS and MS/MS parameters were optimized by infusion experiments using individual working standard solutions of gliotoxin and IS. Full scan data acquisition was performed, and the analyte concentrations were varied to obtain an adequate signal intensity to optimize the individual compound parameters (DP, EP, CEP, CE and CXP) by automatic MRM. The negative ion mode ([M-H]^−^) was found to be the most efficient ionization mechanism for gliotoxin (*m/z* 324.9) and IS (*m/z* 300.8), and at the conclusion of MS/MS optimization, the two most intense fragment signals for each compound were obtained: one for quantification (*m/z* 261.2 for gliotoxin and 151.0 for IS) and the other for peak qualification (*m/z* 243.1 for gliotoxin and 179.0 for IS). The optimization of the source parameters (CUR, CAD, ISV, GS1, GS2 and temperature) was accomplished through flow-injection analysis (FIA) using a mobile phase composed of 50∶50 *v/v* water and acetonitrile/water (95∶5 *v/v*), both containing 1 mM ammonium formate, eluted at 200 µL min^−1^. To develop the HPLC-MS/MS method, a C18 XBridge Shield 150×2.1 mm, 5 µm particle size column was used. Several combinations of methanol, water and acetonitrile were tested for the mobile phase composition; all conditions included 1 mM ammonium formate as an additive. The flow rate (200 to 450 µL min^−1^) and the column oven temperature (25–45°C) were also varied to achieve the optimal run time and peak shape. Under all of the tested conditions, acetonitrile resulted in better ionization of the analytes than methanol. The best overall sensitivity and peak shape were achieved by maintaining the column at 45°C with a mobile phase consisting of a gradient of water and acetonitrile/water 95∶5 *v/v*, both containing 1 mM ammonium formate.

**Figure 3:**
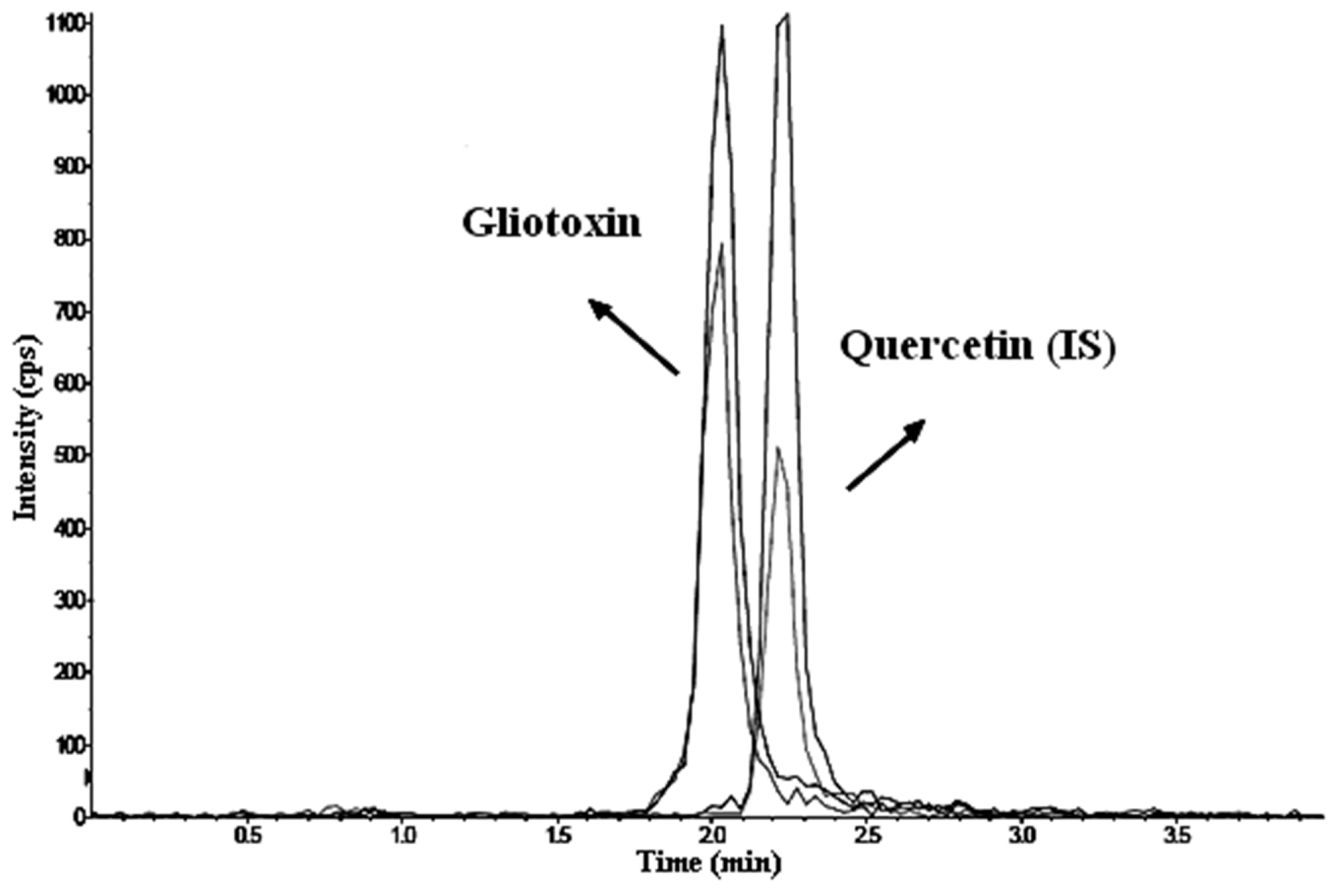
Representative HPLC-MS/MS chromatogram of human serum spiked with standards of gliotoxin and the internal standard quercetin. Ion transitions of gliotoxin: m/z 324.9→261.2 and m/z 324.9→243.1. Ion transitions of quercetin: m/z 300.8→151.0 and m/z 300.8→179.0.

The new HPLC-MS/MS method presents advantages over the other methods that have been described in the literature. For example, the new HPLC-MS/MS method does not produce significant environmental waste because it uses a low flow rate (450 µL min^−1^). The assay is not very time-consuming as the gliotoxin is determined with high selectivity in less than 5 min. Compared to the bioassay based on an automated microplate-reader [15], the new method has higher sensitivity with limits of detection that are six times lower with acceptable accuracy and precision. The present method is also the only one that is fully validated according to modern worldwide regulations, thus ensuring the reliability of the results. A representative chromatogram obtained using the developed method is shown in Figure 3.

### 2. Extraction procedures

To achieve the best recovery of gliotoxin and IS, several procedures for extraction were tested on serum samples, including protein precipitation, liquid-liquid extraction and solid phase extraction. The choice of the most effective procedure was based on the highest recovery and the desired reproducibility. Among the tested procedures, protein precipitation and solid phase extraction were ineffective at extracting gliotoxin and IS from serum. The liquid-liquid extraction method extracted gliotoxin and IS with high efficiency (Figure 2). However, there was a high level of variability in the effectiveness of sample extraction using this procedure for all tested solvents, except for diethyl ether-ethyl acetate (50∶50 *v/v*), which provided an excellent recovery of gliotoxin and IS with low variability.

### 3. Method validation

#### 3.1. Selectivity

**Figure 4:**
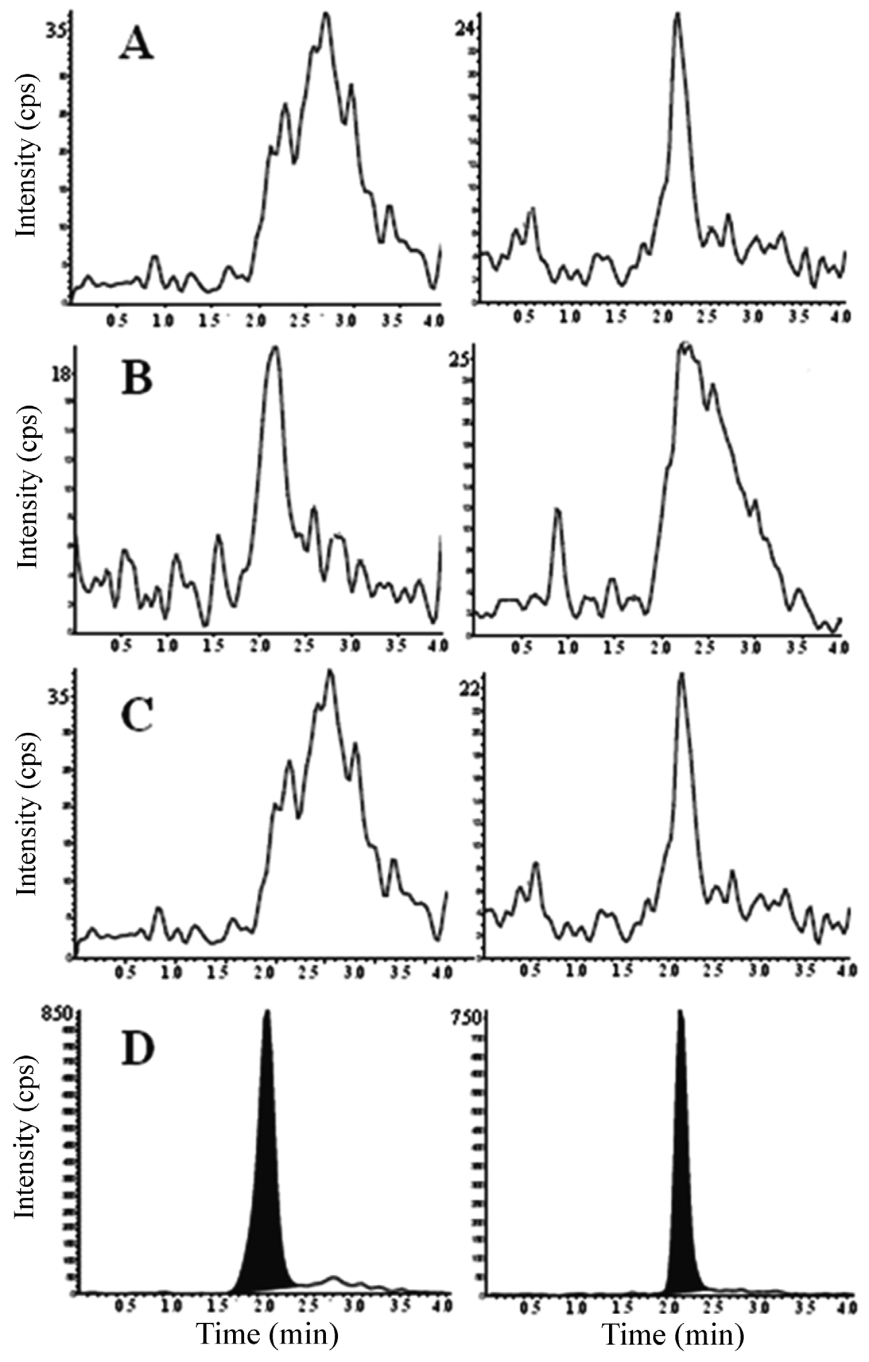
Chromatograms obtained by HPLC-MS/MS for the selectivity study. Negative ion mode: (A) Normal blank serum, (B) lipemic blank serum, (C) hemolyzed blank serum, (D) normal blank serum spiked with IS quercetin (5.0 ng mL^−1^) and gliotoxin at LLOQ level (10.0 ng ml^−1^).

As demonstrated in Figure 4, no additional endogenous interference peaks at the retention times of the analyte and IS were observed. Therefore, the developed method was considered selective.

#### 3.2. Limit of detection (LOD) and lower limit of quantification (LLOQ)

The high sensitivity of the developed method was demonstrated by the low LOD (signal-to-noise ≥3) estimated at 3.0 ng mL^−1^ for gliotoxin. The LLOQ, which was based on a signal-to-noise ratio ≥10 with appropriate precision (RSD<10%), was estimated to be 10.0 ng mL^−1^ for gliotoxin.

#### 3.3. Matrix effect

As demonstrated in Table 2, the variations of the normalized matrix effect (NME) for each compound were less than 15%, indicating that the effects of the biological matrix on the responses of the analytes and IS were insignificant. Therefore, the new method is free of significant matrix interference.

**Table 2:** Variation of the normalized matrix effect (NME) of gliotoxin calculated for assessing matrix effects (n = 8).

Compound	Concentration (ng mL^−1^)	NME	Mean ± S.D.	NME (RSD%)
Gliotoxin	30.0	0.39±0.04	0.42±0.04	10.1
	110.0	0.45±0.06		

Data: Matrix effect calculated by analyte response in matrix/IS response in matrix *versus* analyte response in solution/IS response in solution.

#### 3.4. Linearity

After the three-day assessment, the calibration curves for gliotoxin showed excellent linearity with correlation coefficients (r)>0.99. The means of the individual gliotoxin linear equations and correlation coefficients were y = 0.09443*x*+0.00164 (r = 0.9917). At all concentration levels, the variability in the precision (RSD%) was less than 15% with values ranging from 1.15 to 13.13% for gliotoxin. The individual values for accuracy at each concentration level ranged from 87.35 to 110.96%. These results indicate that this assay provides a reliable response independent of the concentrations utilized.

#### 3.5. Precision, accuracy and sample dilution

The results for accuracy and precision are shown in Table 3. The new method was precise for gliotoxin, with RSD values varying from 4.49 to 7.14% for intra-day and 5.13 to 11.44% for inter-day analyses. The present method also showed satisfactory accuracy, with relative error (RE) values ranging from −0.76 to 8.21 for intra-day and −4.62 to 7.89 for inter-day analyses. These results demonstrate that diluted samples can be analyzed with the same precision and accuracy as undiluted samples. The concentration determined for the diluted samples was 75.36 ng mL^−1^ for gliotoxin (RSD = 7.45%; RE = 7.65%).

**Table 3:** Precision and accuracy of gliotoxin in human serum.

Compound Nme	Control Level	Concentration (ng mL^−1^)	Accuracy Intra-day (RE%)	Accuracy Inter-day (RE%)	Precision Intra-day (RSD%)	Precision Inter-day (RSD%)
	LLOQ	10.0	8.21	7.89	4.49	5.13
	LQC	30.0	3.45	2.07	6.34	8.70
Gliotoxin	MQC	70.0	−1.31	−3.37	7.14	9.83
	HQC	110.0	−0.76	−4.62	5.56	11.44
	DQC	70.0	7.85	7.65	5.53	6.01
Quercetin (IS)	-	5.0	5.86	4.89	4.52	6.25

Data: LLOQ: lower limit of quantification; LQC: low quality control; MQC: medium quality control; HQC: high quality control; DQC: dilution quality control IS: internal standard; RE%: relative error; RSD%: relative standard deviation.

#### 3.6. Carryover test

Using alternating injections of HQC and blank serum samples, no significant interfering peaks were observed at the retention times of the analyte and IS. Therefore, there is no risk of carryover contamination between injections.

#### 3.7 Extraction recovery

The individual extraction recoveries of gliotoxin and IS using liquid-liquid extraction with diethyl ether-ethyl acetate (50∶50 *v/v*) were satisfactory (61.44±2.88% for gliotoxin and 85.15±3.44% for IS) and highly reproducible (RSD<5.0% for gliotoxin and IS). The use of diethyl ether-ethyl acetate (50∶50 *v/v*) is therefore considered effective for the extraction of gliotoxin from human serum.

#### 3.8. Stability assay

Table 4 summarizes the differences between the mean recoveries obtained from the stored and freshly prepared samples. The results show that gliotoxin and IS stock solutions remained stable under refrigeration and freezing conditions. However, the gliotoxin working solutions did not remain stable at room temperature for a period of 6 hours and must therefore be freshly prepared at the time of the experiment. For all of the tested conditions, gliotoxin and IS remained stable in serum.

**Table 4:** Stability data for gliotoxin and quercetin (IS) at different handling and storage conditions (*n* = 6).

Stability			Gliotoxin Level 30.0 ng mL^−1^	Gliotoxin Level 110.0 ng mL^−1^	Quercetin Level 5.0 ng mL^−1^
Working	6 h at room	Recovery %	85.02±3.87	85.99±2.67	98.07±3.42
Solution	temperature	RSD %	4.56	3.11	2.54
Stock	24 hours	Recovery %	101.06±0.56	101.97±5.27	100.36±2.29
Solution	at 4°C	RSD %	5.24	4.70	7.61
	48 hours	Recovery %	101.43±0.56	100.48±6.46	96.48±4.48
	at 4°C	RSD %	5.51	6.41	6.64
	30 days	Recovery %	99.15±6.74	98.44±3.13	99.44±2.75
	at −40°C	RSD %	2.78	3.95	5.05
Serum	Short-term	Recovery %	87.96±4.01	90.74±2.82	99.77±2.88
		RSD %	4.56	3.11	3.65
	Post-processed	Recovery %	97.11±4.37	96.52±0.32	99.54±3.33
		RSD %	4.50	0.33	1.22
	Freeze–thaw	Recovery %	98.86±4.58	95.93±5.86	98.97±2.45
		RSD %	4.64	6.11	2.34
	Long-term	Recovery %	101.5±5.76	95.81±1.87	99.75±2.11
		RSD %	5.68	1.95	1.32

### 4. Application of the analytical method

The developed and validated method was successfully applied to samples from patients suspected of having aspergillosis. All samples were subjected to the ELISA test for galactomannan at the hospital before LC-MS/MS analysis. Among the samples, 10 were positive and 20 were negative for the presence of galactomannans.

Galactomannan is a cell wall polysaccharide released by fungus during growth, including *Aspergillus* species. Currently, ELISA is widely used to diagnose invasive aspergillosis [5], and the results are expressed according to the “galactomannan index” (GMI). Values exceeding the “cutoff” control (GMI = 0.5) are considered positive for galactomannans [4], [19]. ELISA has been considered to be a highly sensitive method (limit of detection at 1 ng mL^−1^ in serum); however, the selectivity of the method is suboptimal, resulting in false-positive results. False reactivity is probably due to several factors. One explanation is gastrointestinal translocation of fungal galactomannan from contaminated food or drink. The wide distribution of galactomannan in the environment does not cause serum reactivity in healthy individuals, but in those with impaired integrity of the intestine (for example newborn babies or patients with mucosal barrier injury due to cytotoxic chemotherapy). Another factor is related to the anti-bacterial therapy using piperacillin/tazobactam and *β*-lactam antibiotics [10], [19]–[21]. Despite its suboptimal selectivity, ELISA is primarily used for screening invasive aspergillosis [4], and for this reason, the results of ELISA were used as a reference for comparing the results obtained by the developed method.

As demonstrated in Table 5, from the samples identified as negative by ELISA (n = 20), three were determined to be positive by the HPLC-MS/MS method. The amounts of gliotoxin were not quantified because they were below the LLOQ. However the presence of gliotoxin in serum of the patients could be an indicative of aspergillosis. Lewis *et al.* (2005) and Puri, *et al.* (2010) also reported the presence of gliotoxin in the sera of patients with invasive aspergillosis and suggested the detection of this mycotoxin as a novel diagnostic approach for invasive aspergillosis [14], [22]. Nevertheless, prospective or case controlled clinical trials must be conducted to demonstrate that the presence of gliotoxin in serum means that a patient has invasive aspergillosis.

**Table 5:** Serum gliotoxin concentrations in patients at risk for aspergillosis.

	Sample	Origin	Presence of gliotoxin by LC-MS/MS
	1	BMT	ND
	2	CT	ND
	3	BMT	ND
	4	BMT	ND
	5	BMT	ND
	6	BMT	ND
**NEGATIVE FOR**	7	BMT	<10.0 ng mL^−1^
**GALACTOMANAN**	8	BMT	ND
**(ELISA)**	9	BMT	ND
	10	BMT	<10.0 ng mL^−1^
	11	BMT	ND
	12	BMT	<10.0 ng mL^−1^
	13	BMT	ND
	14	BMT	ND
	15	BMT	ND
	16	BMT	ND
	17	BMT	ND
	18	BMT	ND
	19	BMT	ND
	20	BMT	ND
	1	CT	138.5 ng mL^−1^
	2	BMT	ND
	3	CT	<10.0 ng mL^−1^
**POSITIVE FOR**	4	CT	<10.0 ng mL^−1^
**GALACTOMANAN**	5	BMT	35.0 ng mL^−1^
**(ELISA)**	6	CT	ND
	7	CT	ND
	8	BMT	ND
	9	BMT	ND
	10	BMT	<10.0 ng mL^−1^

Data: BMT : bone marrow transplant; CT: chemotherapy; ND: not detected.

From the positive samples tested by ELISA (n = 10), five were found to positive for gliotoxin using the HPLC-MS/MS method. Among these samples, three were below the LLOQ (<10 ng mL^−1^), one contained 35.0 ng mL^−1^ of gliotoxin and one showed levels of gliotoxin above the HQC and was re-analyzed using the validated dilution procedure (item 2.6.6, p.11), resulting in a final sample concentration of 138.5 ng mL^−1^.

The remaining five samples identified as positive via ELISA analysis were found to be negative by HPLC-MS/MS. ELISA is a suboptimal method and may suffer from interference from various other components present in serum [10], [23]. The use of the method developed in this report could reduce this interference because multiple reaction monitoring (MRM) was able to detect gliotoxin and its specific fragments in the analyzed matrices.

### Conclusion

The new, reproducible, selective and fast HPLC-MS/MS method developed in this study was found to be suitable for the quantification of gliotoxin in human serum. The method is linear, precise, accurate and free of matrix interferences. Stability studies showed that working solutions of gliotoxin should be prepared immediately before use. Analysis of samples from immunocompromised patients supports the applicability of this technique. Detection of gliotoxin by this method may facilitate early diagnosis of invasive aspergillosis.
